# Medically unexplained symptoms and experiences with healthcare among emerging adults exposed to multiple types of potentially traumatic events

**DOI:** 10.1371/journal.pone.0310335

**Published:** 2024-09-09

**Authors:** Caterina Obenauf, Gina P. Owens, Sam DeHart

**Affiliations:** Department of Psychology, University of Tennessee, Knoxville, TN, United States of America; University of Health Sciences, Beyhekim Training and Research Hospital, TÜRKIYE

## Abstract

Experiencing multiple types of traumatic events can increase the risk of developing somatic and posttraumatic stress symptoms (PTSS). Medically unexplained symptoms (MUS), or somatic symptoms that lack a distinct medical explanation, often coexist with PTSS in emerging adults and may be due to common underlying mechanisms. Coping strategies have been associated with PTSS, but have not been studied in trauma-exposed individuals with MUS. The current study examined the relationship between the number of types of potentially traumatic events experienced and MUS among emerging adults, considering the influence of PTSS and engagement and disengagement coping. A sample of 363 emerging adults (Mean = 18.91) completed self-report measures of trauma history, PTSS, MUS, experiences with healthcare providers, and coping strategies. Dissatisfaction with healthcare providers was reported by 11.3% of participants reporting MUS, with over half (52.8%) feeling their concerns were dismissed. Hierarchical linear regression showed that the number of types of traumatic events experienced did not predict MUS after accounting for PTSS. Moderation hypotheses linking traumatic events and coping strategies were not supported. Results suggest that PTSS explains the relationship between exposure to different traumatic events and MUS. Findings may have diagnostic and treatment implications for healthcare providers working with emerging adults who have experienced trauma.

## Introduction

Between 10% and 24% of emerging adults, a life stage approximately between the ages of 18 to 25, exhibit persistent somatic symptoms lacking a clear medical explanation, termed medically unexplained symptoms (MUS) [1]. MUS are often attributed to functional somatic syndromes like fibromyalgia, chronic widespread pain, chronic fatigue syndrome, temporomandibular disorder, and irritable bowel syndrome, which are all characterized by symptoms without discernible biological or physical causes and lacking consensus on diagnostic criteria [2]. These syndromes share both somatic (e.g., pain, fatigue) and psychological symptoms (e.g., anxiety, depression, posttraumatic stress) [3,4]. Given the increased risk of psychiatric disorders during emerging adulthood, exploring the interplay between psychopathology and somatic concerns which are prevalent in college students is imperative [5,6].

Understanding the biopsychosocial mechanisms underlying MUS is crucial for providing effective treatment. Psychological trauma, particularly the number and types of potentially traumatic events (PTEs) experienced, is a possible mechanism for MUS etiology, with experiencing many PTEs potentially leading to chronic stress responses, and subsequently, somatic symptoms [3,7,8]. Trauma-exposed individuals, especially those experiencing multiple traumatic events, are two to three times more likely than the general population to have a functional somatic syndrome diagnosis, indicating a potential link between coping styles and somatization [2]. The prevalence of functional somatic syndromes in trauma-exposed emerging adults is unknown. Exploring the relationship between trauma and MUS in this population is crucial, however, given the heightened risk for psychiatric disorders in emerging adulthood [6].

Posttraumatic stress symptoms (PTSS), encompassing re-experiencing, avoidance, negative cognitions, and arousal, often follow trauma exposure [9]. Examining both PTSS and MUS among trauma-exposed emerging adults is essential, given the established overlap in coping factors contributing to their etiology [10,11]. While research has examined functional somatic syndromes and related physical health outcomes in association with PTSS and trauma exposure [12,13], to our knowledge, no studies have examined MUS, PTSS, and coping styles that contribute to these outcomes among trauma-exposed emerging adults. Despite trauma exposure being linked to MUS and functional somatic syndromes, studies on the associations between PTSS severity and MUS are limited and often underpowered [2,14]. Moreover, coping strategies may vary among individuals with MUS, highlighting the importance of understanding coping mechanisms for tailored interventions after PTEs [15].

Two broad coping strategies have been identified for traumatic events: disengagement and engagement coping. Disengagement coping, involving methods like problem avoidance and social withdrawal, initially reduces distress but contributes to the development and maintenance of PTSS over time [16]. Disengagement coping is positively associated with PTSS severity in survivors of various trauma types, and more severe somatic symptoms are linked to greater use of disengagement coping in adolescents with abdominal pain, chronic pain, and somatic complaints [17,18]. Conversely, engagement coping strategies like acceptance, positive thinking, and cognitive restructuring are associated with fewer somatic, anxiety, and depression symptoms [19]. Despite these findings, the impact of engagement and disengagement coping strategies on comorbid MUS and PTSS after PTE exposure among emerging adults is unknown. Coping strategies have been found to moderate the relationship between PTEs and PTSS, with disengagement and engagement coping linked to higher and lower PTSS levels respectively [20]. While trauma is recognized as a potential etiological factor in MUS development [8], no studies have explored the potential moderating effects of coping style on MUS in relation to PTSS. Mixed findings have been found on whether coping strategies moderate the relationship between early life and current stressors and somatic symptoms [21]. One study with a college student sample found that after controlling for depressive symptoms, negative cognitive coping strategies no longer moderated the relation between current stressors and current somatic symptom severity; those findings suggest that psychiatric symptoms play a role in the relation between stress and somatic symptoms, but this has yet to be studied among individuals with PTSS and exposure to PTEs [22]. Given coping style’s moderating effect on PTE and PTSS, coupled with the comorbidity between PTSS and MUS, coping style may also moderate the relationship between PTEs and MUS. The potential role of PTSS in this relation is unclear and will be explored in the current study.

### The current study

The current study explores the relations among the number of PTE types experienced, MUS, posttraumatic stress symptoms (PTSS), and coping strategies in emerging adults. Given the unclear relationship between the number of PTE types and MUS and the high comorbidity of PTSS and MUS, our study aims to clarify this relationship and provide insights into potential contributing mechanisms. Additionally, we investigate the role of coping strategies in the relationship between PTE exposure and MUS. Engagement and disengagement coping strategies are moderating factors between PTE exposure and PTSS [20], however it is unknown whether they also play a role in the relationship between PTE exposure and MUS. Given the frequent comorbidity of PTSS and MUS, we will explore how engagement and disengagement coping strategies interact with PTE exposure, PTSS, and MUS to illuminate mechanisms that may either lessen or strengthen the association between PTE exposure and MUS. In the current study, we first hypothesized that the number of PTE types experienced would be related to MUS after controlling for the contribution of PTSS (Hypothesis 1). Second, we hypothesized that this relation would be stronger among those who report higher use of disengagement coping strategies and lower use of engagement coping strategies (Hypothesis 2).

## Method

### Participants

Participants (*N* = 363) were students attending a large state university who reported experiencing one or more PTEs. The mean age of our sample was 18.91 years (*SD* = 2.23). The race and ethnicity distribution of our sample was as follows: White (79.8%), Asian and/or Pacific Islander (4.1%), Hispanic/Latino/a/x (4.1%), Black (3.8%), Middle Eastern/North African (1.4%), Native American (0.3%), Biracial/Multiracial/Multiethnic (5.5%), and additional identity or prefer not to answer (0.8%). Most participants reported being single (80.9%), with 17.4% in a long-term relationship and 1.4% married. Many participants reportedly came from high income households ($75,000 or more per year, 51.0%). Most participants identified as cisgender woman (81.2%), followed by cisgender man (10.6%), other (4.1%), non-binary (2.5%), questioning (0.5%), and transgender man (0.3%). Over half of our sample reportedly experienced three or more types of traumatic events (59.1%), with 18.2% reporting one type of event and 23.1% experiencing two types of events. The most common PTEs experienced were sudden death of a loved one (64.3%), a natural disaster (42.7%), and seeing someone die suddenly or get badly hurt or killed (25.9%). Almost a third of the sample (30.9%) met criteria for probable posttraumatic stress disorder (PTSD) [23,24]. Much of the sample reported medium (41.6%) or high severity (15.2%) of MUS. Many participants (57.6%) reported talking to their healthcare provider about their endorsed somatic symptoms. Of these participants, 79% indicated being diagnosed with a functional somatic syndrome; common diagnoses included irritable bowel syndrome, premenstrual syndrome or premenstrual dysphoric disorder, and tension headache or persistent facial pain.

### Measures

#### Trauma history screen

The Trauma History Screen (THS) [25] is a 13-item scale that asks participants to select “yes” or “no” to whether they have experienced a given traumatic event. Participants who choose “yes” are prompted to indicate how many times they have experienced that particular event. A sample event includes, “attack with a gun, knife, or weapon.” Endorsing one or more traumatic events is considered indicative of trauma exposure. The THS is a valid measure of trauma exposure and has been shown to be comparable or better than other, longer measures of trauma exposure, such as the Trauma History Questionnaire [26], with high test-retest reliability among college students [25].

#### PTSD checklist for DSM-5 with Criterion A

The PTSD Checklist for DSM-5 (PCL-5) with Criterion A [24] assesses for PTSD symptom severity endorsed after a Criterion A traumatic event. Participants first complete several questions that assess whether their endorsed stressful event is a Criterion A trauma (e.g., Did it involve actual or threatened death, serious injury, or sexual violence?). Next, participants complete 20 items that ask about the severity of their symptoms due to their endorsed traumatic event. Items include: “Repeated, disturbing, and unwanted memories of the stressful experience?” Responses are scored on a 5-point Likert scale from 0 (not at all) to 4 (extremely), and the responses for each item are summed to generate a total score of PTSD symptom severity. A score of 30 or higher on this scale is considered a probable PTSD diagnosis [23]. Among college student samples, the PCL-5 has good internal consistency (α = .94) and test-retest reliability (*r* = .82). Convergent and discriminant validity have been supported [26].

#### Patient health questionnaire

The Patient Health Questionnaire– 15 (PHQ-15) [27] is a 15-item questionnaire that assesses the severity of somatic symptoms (e.g., back pain, dizziness) during the past four weeks. Items are rated on a Likert scale from 0 (not bothered at all) to 2 (bothered a lot). Among college students, the PHQ-15 demonstrated high internal consistency across cultures (Cronbach’s α = 0.80–0.86; [27,28]. The PHQ-15 is a valid and reliable questionnaire for individuals at risk of somatoform disorder, or functional somatic syndromes, with 21% of primary care patients endorsing 3 or more somatic symptoms on the PHQ-15 (score ≥ 6) also having a functional somatic syndrome diagnosis, with sensitivity of 78% and specificity at 71% for a cut off score of ≥ 3 [29].

#### Additional questions for assessing medically unexplained symptoms

One limitation of the PHQ-15 is that it does not distinguish between medically explained and unexplained symptoms, which typically requires a clinical diagnostic interview. After administering the PHQ-15, participants were asked a series of questions similar to previous studies to interpret whether symptoms expressed in the PHQ-15 were unexplained [30]. We asked: “have you talked to your healthcare provider about some of these problems?” with answer options of “yes” and “no.” Participants who said yes were asked for satisfaction with care, if they felt their provider handled their concerns well, and if they agreed with their provider’s diagnoses or explanations for their concerns. Participants were also asked if they felt their provider dismissed their problems, had diagnostic tests, and went to see their provider or multiple providers several times. We additionally asked participants if they have been diagnosed with any functional somatic syndromes commonly attributed to having no medically explained cause (e.g., somatoform disorder, irritable bowel syndrome, fibromyalgia).

### Coping strategies inventory

The Coping Strategies Inventory (CSI) [31] is a 72-item measure that assesses for coping strategies utilized in response to stressors and has been used in trauma research [32]. Responses are on a 5-point Likert scale where participants determine the extent to which they used a given strategy in handling their chosen traumatic event, from 0 (not at all) to 4 (very much). Two tertiary factors can be calculated, engagement and disengagement coping, and these were the focus of the current study. A sample item for engagement coping includes, “I let my emotions out.” A sample item for disengagement coping includes, “I blamed myself.” The CSI has been found to have acceptable test-retest reliability, and the factors on the CSI map onto factors on other measures of coping strategies [31]. Among studies with trauma-exposed participants, the CSI has demonstrated high internal consistency reliability ranging from .89 to .92 [32,33].

### Procedure

Participants enrolled in introductory psychology classes were recruited from the department of psychology research pool at a large university in the southeastern United States. Prior to beginning the survey, individuals reviewed an informed consent document. Participants indicated their written consent to participate by clicking yes to continue to the online survey. Participants had to be 18 years old or older to participate and endorse at least one traumatic event on the Trauma History Screen [25]. Eligible participants then completed a demographic questionnaire, as well as self-report questionnaires described below. Participants received research participation credit after completion of the survey. Study procedures and analyses were reviewed and approved by the University of Tennessee-Knoxville Institutional Review Board and were conducted according to the principles of the Declaration of Helsinki. The approval number is UTK IRB-22-06983-XP. Data were collected between July 7, 2022 and October 12, 2022. The data that support the findings of this study are available on Open Science Framework.

### Statistical analyses

Analyses in this study were conducted using SPSS software (version 27.0). Post-hoc analyses on G*Power [34] indicated .99 power for the current study to detect a small to medium effect size (*f*^2^ *=* .15). We calculated means, standard deviations, and internal consistency reliability estimates for all continuous variables. Skewness and kurtosis statistics were less than ± 1.0 and there was no evidence of multicollinearity of independent variables (all correlations < .8), thus the data met assumptions of hierarchical linear regression. While 470 individuals consented to the survey, some participants were removed from final analyses due to not completing the survey in its entirety (*n* = 75), failing attention checks (*n* = 21), or missing three or more items on a single measure (*n* = 11). An absence of discernible patterns of missing data indicated a random distribution of missing values in the dataset. When less than 5% of the data are missing, most methods of handling missing data are considered appropriate [35]; thus, the mean substitution method was used to handle missing data for measures that were missing one or two items.

Bivariate correlations were conducted to test the associations between number of PTE types experienced and severity of MUS and PTSS (Table 1). Moderation analyses focused on interactions (Number of PTE Types x Engagement Coping, Number of PTE Types x Disengagement Coping) in a hierarchical linear regression model with MUS as the outcome to test Hypotheses 1 and 2. The model included the number of types of PTEs experienced in the first step as the predictor, followed by controlling for PTSS in the second step. Evaluating the strength of the relationship between the number of types of PTEs experienced and MUS after controlling for PTSS allowed us to test Hypothesis 1. Then the model was followed by the addition of engagement and disengagement coping in the third step. To evaluate potential moderating effects, interaction terms were mean-centered prior to analysis. We examined whether the inclusion of the interaction terms statistically significantly increased R^2^, which would indicate if moderation had occurred. This approach allowed for exploration of differences in the impact of the number of types of PTEs experienced on MUS based on varying levels of both engagement and disengagement coping. Cohen’s f^2^ was calculated to evaluate the effect size of the predictor variables.

**Table 1:** Range, means, standard deviations, and correlations among variables (*N* = 363).

Measure	Cronbach’s alpha	Range	Mean	SD	Number of PTE Types	PTSS	MUS Severity	Engagement Coping
Number of Potentially Traumatic Event (PTE) Types	--	1–14	3.51	2.41	--	--	--	--
Posttraumatic Stress Severity (PTSS)	.93	0–69	22.34	16.06	.298***	--	--	--
Medically Unexplained Symptom (MUS)Severity	.84	0–28	9.26	5.67	.224***	.523***	--	--
Engagement Coping	.93	1–113	51.01	22.97	-.008	.192***	.063	--
Disengagement Coping	.92	1–126	55.75	26.09	.221***	.630***	.375***	.115*

*Note*. *p < .05.

**p < .01.

***p < .001.

## Results

The ranges, means, standard deviations, and correlations among all study variables are in Table 1. Significant positive correlations were observed between PTSS severity and greater number of PTE types experienced (*p* < .001), engagement coping (*p* < .001), disengagement coping (*p* < .001), and MUS severity (*p* < .001). Significant positive correlations were also observed between MUS severity and greater number of PTE types experienced (*p* < .001), as well as disengagement coping *(p* < .001). Disengagement coping and engagement coping were also positively correlated (*p* = .028).

Among the 57.8% of participants who had spoken to healthcare providers about their endorsed somatic symptoms, some (11.3%) reported dissatisfaction with how their healthcare provider handled their concerns. While most participants agreed or somewhat agreed with their provider’s diagnosis or handling of symptoms, several participants (26.0%) somewhat or completely disagreed. Over half of participants (52.8%) reportedly felt that at least one of their providers dismissed their concerns. Many participants reported having more than one diagnostic test or examination for their endorsed symptoms (40.3%). Regarding help-seeking behaviors, 31.1% of participants reported going to several providers because of their symptoms, 14.6% saw their provider several times, and 14.6% engaged in both behaviors. Many participants (57.1%) reported that their provider attributed their symptoms to some non-organic cause (e.g., anxiety (*n* = 58), stress (*n* = 36), and depression (*n* = 31)). In fewer instances, providers attributed symptoms to trauma history (*n* = 5).

Hypothesis 1 was tested using hierarchical linear regression with MUS severity as the outcome (Table 2). While Hypotheses 1 and 2 were not supported, the overall regression model predicting MUS severity was significant, explaining 27.8% of the variance, F(6, 339) = 23.11, *p* < .001, Adj. R^2^ = .278. A greater number of types of PTEs experienced significantly predicted more severe MUS severity in step 1 (β = .222, f^2^ = .05, *p* < .001, 95% Confidence Interval [.28, .76]) with a small effect size, F(1, 344) = 17.90, *p* < .001, Adj. R^2^ = .047. Adding PTSS severity resulted in more severe PTSS significantly predicting more severe MUS (β = .505, f^2^ = 43, *p* < .001, 95% Confidence Interval [.14, .21]) with a large effect size, but a greater number of types of PTEs experienced no longer significantly predicted more severe MUS in step 2 (β = .072, f^2^ = 36, *p* = .132, 95% Confidence Interval [-.05, .39]) with a large effect size, F(2, 343) = 67.462, *p* < .001, ΔR^2^ = .231. In step 3, adding engagement coping (β = -.049, f^2^ = 08, *p* = .296, 95% Confidence Interval [-.03, .04]) and disengagement coping (β = .069, f^2^ = 08, *p* = .246, 95% Confidence Interval [-.01, .04]) did not significantly improve the model with small effect sizes (*p* = .28).

**Table 2:** Moderation analyses predicting medically unexplained symptoms.

	Medically Unexplained Symptoms^a^					
Predictors	B	SE	β	t	*p*	95% Confidence Interval
Step 1						
Number of Potentially Traumatic Event (PTE) Types	.516	.122	.222	4.230	< .001	[.28, .76]
Step 2						
Number of PTE Types	.168	.111	.072	1.509	.132	[-.05, .39]
Posttraumatic Stress Symptoms (PTSS)	.175	.017	.505	10.549	< .001	[.14, .21]
Step 3						
Number of PTE Types	.153	.112	.066	1.372	.171	[-.07, .37
PTSS	.164	.021	.473	7.767	< .001	[.12, 21]
Engagement Coping	-.012	.011	-.050	-1.078	.282	[-.03, .01]
Disengagement Coping	.015	.013	.071	1.208	.228	[-.01, .04]
Step 4						
Number of PTE Types	.141	.113	.061	1.246	.214	[-.08, .36]
PTSS	.164	.021	.472	7.732	< .001	[.12, .21]
Engagement Coping	-.012	.011	-.049	-1.047	.296	[-.03, .04]
Disengagement Coping	.015	.013	.069	1.162	.246	[-.01, .04]
Number of PTE Types x Engagement Coping	-.003	.004	-.028	-.603	.547	[-.01, .01]
Number of PTE Types x Disngagement Coping	.004	.004	.047	1.012	.312	[-.00, .01]

Note. *p < .05

** *p* < .01

*** *p* < .001.

^a^Adj. R^2^ = .047, ΔR^2^ Step 2 = .231, ΔR^2^ Step 3 = .001, ΔR^2^ Step 4 = -.001.

The final step of the hierarchical linear regression analysis included the interaction terms (Number of PTE Types x Engagement Coping, Number of PTE Types x Disengagement Coping) to explore whether the relationship between the number of PTE types and medically unexplained symptoms (MUS) severity was moderated by coping strategies. Inclusion of both interaction terms (Number of PTE Types x Engagement Coping, Number of PTE Types x Disengagement Coping) in step four did not significantly improve the model (*p* = .54); thus, neither engagement nor disengagement coping significantly changed the relation between number of PTE types on MUS severity with small effect sizes (f^2^ = .08 for both terms). Given the lack of significant interaction terms, we did not conduct further analyses to compare the effects of engagement and disengagement coping on MUS severity.

## Discussion

The current study explored the relationship between the number of types of PTEs experienced, PTSS severity, engagement and disengagement coping, and MUS severity in a sample of emerging adults in a large public university in the United States who reported experiencing trauma. The findings revealed several noteworthy associations and provided insights into the complex interplay of these variables. Additionally, some participants in the sample reported that their healthcare providers often attributed their somatic symptoms to stress, anxiety, or other non-organic causes, and several felt their concerns were dismissed.

A high prevalence of trauma exposure in the sample, with a substantial proportion of participants reporting multiple PTEs. This finding aligns with existing research highlighting the pervasive nature of PTEs among emerging adults [36]. Notably, a substantial number of participants also met the criteria for probable PTSD, further emphasizing the significance of trauma in this population.

Regarding MUS, a substantial portion of participants reported medium to high severity of somatic symptoms. This result is consistent with previous research demonstrating the prevalence of MUS among trauma survivors [2,12]. Additionally, a significant number of participants discussed their somatic symptoms with healthcare providers, and some reported dissatisfaction with their healthcare experiences, indicating potential gaps in addressing the needs of emerging adults with MUS in clinical settings. The findings also highlighted the diverse attributions made by healthcare providers regarding the causes of these symptoms, with anxiety and stress being the most common factors. This variability in provider attributions underscores the complexity of diagnosing and managing MUS.

Contrary to Hypothesis 1, which posited that a greater number of PTEs experienced would predict more severe MUS, our findings did not support a direct relationship between the number of types of PTEs experienced and MUS severity once PTSS severity was accounted for. Instead, PTSS severity emerged as a robust predictor of MUS severity. This finding aligns with the literature on the comorbidity of PTSD and somatic symptoms [12,13], suggesting that individuals with more severe PTSD symptoms are more likely to experience heightened somatic complaints. This also adds to previous theoretical conceptualizations of MUS, which suggest that psychological trauma could be a contributing factor in the development and severity of these symptoms [7]. Importantly, this relationship highlights the need for clinicians to consider both psychological and physical symptomatology when working with trauma-exposed individuals.

In addition to the unexpected findings above, the current study had null results regarding the potential moderating effects of engagement and disengagement coping on the relationship between the number of types of traumatic events experienced and MUS severity. Further, engagement and disengagement coping strategies did not contribute to MUS severity after considering PTSS severity. The inclusion of these interaction terms did not significantly improve the model, indicating that coping strategies did not change the nature of the relationship between PTE exposure and MUS severity in this sample. This is in line with a previous qualitative study that similarly found that participants with comorbid depressive symptoms and somatic symptoms reported engagement and disengagement coping strategies to be ineffective in managing MUS [37]. These unexpected results suggest that, at least within the context of this study, the impact of PTEs on somatic symptom severity may not vary significantly based on employed coping strategies.

Several limitations should be considered when interpreting the results of this study. First, the analyses of the current study were cross-sectional and correlational in design, thus limiting the ability to draw causal conclusions. Longitudinal and experimental research designs could further illuminate the temporal order of the variables in our study, as the temporal order between psychological distress and MUS has not been established [38]. Future studies could consider longitudinally following individuals exposed to trauma to elucidate the emergence of MUS; the utility of exploring biomarkers for stress and their relationship with MUS would greatly add to the understanding of the relationship between trauma and MUS. Second, the participants in this study were mostly white women emerging adults working towards receiving an undergraduate degree. The results may not generalize to other groups with different race, ethnic background, gender, and socioeconomic status. Additionally, given that participants self-selected to participate in a study related to trauma, their results may differ from others who are not as willing to disclose past experiences [39]. The design of the current study relied on self-report measures, which introduces the potential for response biases given that there was no third-party verification of self-reported experiences and symptoms.

Despite these limitations, our results have implications for healthcare providers working with emerging adults with histories of trauma who fit within the demographics of the current study. Based on our findings, it is crucial for healthcare providers to consider trauma as a potential factor contributing to physical symptoms and consider referrals for behavioral health services. A small portion of our participants reported their healthcare provider attributed their symptoms to their trauma history. Most participants reported that their healthcare provider attributed their symptoms to stress and anxiety without presumably assessing their trauma history. A review [40] of the experiences of patients when communicating with their providers highlighted the importance of healthcare providers being sensitive to specific factors like a patient’s identities (e.g., not being paternalistic because of a patient’s young age) and considering psychosocial aspects related to a patient’s presenting concerns. These findings, along with the findings of the current study, highlight the need for improved training for healthcare providers in assessing trauma history and responding to trauma survivors’ concerns about their physical symptoms in a trauma-informed fashion. Integrating trauma-informed care approaches, such as routine screening for traumatic experiences and providing appropriate referrals to mental health services, may enhance the care for individuals with MUS [41]. Additionally, policies in healthcare facilities should support interdisciplinary care models that address both physical and psychological aspects of health [42]. Given the findings of this study emphasizing the overlap between PTSS and MUS, such comprehensive care approaches through formally establishing collaboration among healthcare providers, mental health professionals, and social services create a holistic approach to patient care. By improving provider awareness and response to trauma, we can promote better physical and mental health outcomes among emerging adults exposed to multiple traumatic events.

## References

[1] Brown RJ. Introduction to the special issue on medically unexplained symptoms: background and future directions. Clinical Psychology Review. 2007;27(7):769–780. doi: 10.1016/j.cpr.2007.07.003 17707564

[2] Afari N, Ahumada SM, Wright LJ, Mostoufi S, Golnari G, Reis V, et al. Psychological trauma and functional somatic syndromes: a systematic review and meta-analysis. Psychosomatic Medicine. 2014;76(1):2. doi: 10.1097/PSY.0000000000000010 24336429 PMC3894419

[3] Roelofs K., Spinhoven P. Trauma and medically unexplained symptoms: Towards an integration of cognitive and neuro-biological accounts. Clinical Psychology Review. 2007;27(7):798–820. doi: 10.1016/j.cpr.2007.07.004 17728032

[4] Russo J, Katon W, Sullivan M, Clark M, Buchwald D. Severity of somatization and its relationship to psychiatric disorders and personality. Psychosomatics. 1994;35(6):546–556. doi: 10.1016/S0033-3182(94)71723-0 7809357

[5] El Ansari W, Oskrochi R, Stock C. Symptoms and health complaints and their association with perceived stress: Students from seven universities in England, Wales and Northern Ireland. Journal of Public Health. 2013;21:413–425. doi: 10.1007/s10389-013-0571-x

[6] Henin A, Berman N. The promise and peril of emerging adulthood: Introduction to the special issue. Cognitive and Behavioral Practice. 2016;23(3):263–269. doi: 10.1016/j.cbpra.2016.05.005

[7] Brown RJ. Medically unexplained symptoms: a new model. Psychiatry. 2006;5(2):43–47. doi: 10.1383/psyt.2006.5.2.43

[8] Kolassa IT, Ertl V, Eckart C, Kolassa S, Onyut LP, Elbert T. Spontaneous remission from PTSD depends on the number of traumatic event types experienced. Psychological Trauma: Theory, Research, Practice, and Policy. 2010;2(3):169. doi: 10.1037/a0019362

[9] Ginzburg K, Ein-Dor T, Solomon Z. Comorbidity of posttraumatic stress disorder, anxiety and depression: A 20-year longitudinal study of war veterans. Journal of Affective Disorders. 2010;123(1–3):249–257. doi: 10.1016/j.jad.2009.08.006 19765828

[10] Beck JE. A developmental perspective on functional somatic symptoms. Journal of Pediatric Psychology. 2008;33(5):547–562. doi: 10.1093/jpepsy/jsm113 18056142

[11] Steinhausen HC. Developmental psychopathology in adolescence: findings from a Swiss study–the NAPE Lecture 2005. Acta Psychiatrica Scandinavica. 2006;113(1):6–12. doi: 10.1111/j.1600-0447.2005.00706.x 16390363

[12] Pacella ML, Hruska B, Delahanty DL. The physical health consequences of PTSD and PTSD symptoms: a meta-analytic review. Journal of Anxiety Disorders. 2013;27(1):33–46. doi: 10.1016/j.janxdis.2012.08.004 23247200

[13] Sharp TJ, Harvey AG. Chronic pain and posttraumatic stress disorder: mutual maintenance? Clinical Psychology Review. 2001;21(6):857–877. doi: 10.1016/s0272-7358(00)00071-4 11497210

[14] Romans S, Cohen M. Unexplained and underpowered: the relationship between psychosomatic disorders and interpersonal abuse—a critical review. Harvard Review of Psychiatry. 2008;16(1):35–54. doi: 10.1080/10673220801933788 18306098

[15] Roohafza H, Keshteli AH, Daghaghzadeh H, Afshar H, Erfani Z, Adibi P. Life stressors, coping strategies, and social supports in patients with irritable bowel syndrome. Advanced Biomedical Research. 2016; *5*. doi: 10.4103/2277-9175.190935 27761433 PMC5070037

[16] Taft CT, Resick PA, Panuzio J, Vogt DS, Mechanic MB. Coping among victims of relationship abuse: A longitudinal examination. Violence and Victims. 2007;22(4):408–418. doi: 10.1891/088667007781553946 17691549 PMC2977524

[17] Batchelder AW, Safren SA, Coleman JN, Boroughs MS, Thiim A, Ironson GH, et al. Indirect effects from childhood sexual abuse severity to PTSD: The role of avoidance coping. Journal of Interpersonal Violence. 2021;36(9–10):NP5476–NP5495. doi: 10.1177/0886260518801030 30246600 PMC6785355

[18] Compas BE, Boyer MC, Stanger C, Colletti RB, Thomsen AH, Dufton LM, et al. Latent variable analysis of coping, anxiety/depression, and somatic symptoms in adolescents with chronic pain. Journal of Consulting and Clinical Psychology. 2006;74(6):1132–1142. doi: 10.1037/0022-006X.74.6.1132 17154742

[19] Thomsen AH, Compas BE, Colletti RB, Stanger C, Boyer MC, Konik BS. Parent reports of coping and stress responses in children with recurrent abdominal pain. Journal of Pediatric Psychology. 2002;27(3):215–226. doi: 10.1093/jpepsy/27.3.215 11909929

[20] Elzy M, Clark C, Dollard N, Hummer V. Adolescent girls’ use of avoidant and approach coping as moderators between trauma exposure and trauma symptoms. Journal of Family Violence. 2013;28:763–770. doi: 10.1007/s10896-013-9546-5

[21] Kuhar M, Zager Kocjan G. Adverse childhood experiences and somatic symptoms in adulthood: A moderated mediation effects of disturbed self-organization and resilient coping. Psychological Trauma: Theory, Research, Practice, and Policy. 2022;14(8):1288–1298. doi: 10.1037/tra0001040 34166046

[22] Garner MJ. Stress and somatic symptoms: Rumination and negative affect as moderators. [Dissertation]. Seattle Pacific University; 2016.

[23] Blevins CA, Weathers FW, Davis MT, Witte TK, Domino JL. The posttraumatic stress disorder checklist for DSM‐5 (PCL‐5): Development and initial psychometric evaluation. Journal of Traumatic Stress. 2015;28(6):489–498. doi: 10.1002/jts.22059 26606250

[24] Weathers FW, Litz BT, Keane TM, Palmieri PA, Marx BP, Schnurr PP. The PTSD Checklist for DSM-5 (PCL-5). *Scale available from the National Center for PTSD at www.ptsd.va.gov*. 2013;10(4):206.

[25] Carlson EB, Smith SR, Palmieri PA, Dalenberg C, Ruzek JI, Kimerling R, et al. Development and validation of a brief self-report measure of trauma exposure: the Trauma History Screen. Psychological Assessment. 2011;23(2):463–477. doi: 10.1037/a0022294 21517189 PMC3115408

[26] Hooper LM., Stockton P, Krupnick JL, Green BL. Development, use, and psychometric properties of the Trauma History Questionnaire. Journal of Loss and Trauma. 2011;16(3), 258–283. doi: 10.1080/15325024.2011.572035

[27] Kroenke K, Spitzer RL, Williams JB. The PHQ-15: Validity of a new measure for evaluating the severity of somatic symptoms. Psychosomatic Medicine. 2002;64(2):258–266. doi: 10.1097/00006842-200203000-00008 11914441

[28] Martinez-Lorca A, Martinez-Lorca M, Criado-Álvarez JJ, García JA, Armesilla MDC. Psychometric Properties of the Patient Health Questionnaire-15 (PHQ-15) and the General Health Questionnaire-28 (GHQ-28). Validation in Spanish university students during COVID-19 outbreak. International Journal of Psychology and Psychoanalysis. 2021;7(1). doi: 10.23937/2572-4037.1510049

[29] van Ravesteijn H, Wittkampf K, Lucassen P, van de Lisdonk E, van den Hoogen H, van Weert H, et al. Detecting somatoform disorders in primary care with the PHQ-15. The Annals of Family Medicine. 2009;7(3):232–238. doi: 10.1370/afm.985 19433840 PMC2682971

[30] Steinbrecher N, Koerber S, Frieser D, Hiller W. The prevalence of medically unexplained symptoms in primary care. Psychosomatics. 2011;52(3):263–271. doi: 10.1016/j.psym.2011.01.007 21565598

[31] Tobin DL, Holroyd KA, Reynolds RV, Wigal JK. The hierarchical factor structure of the Coping Strategies Inventory. Cognitive Therapy and Research. 1989;13:343–361. doi: 10.1007/BF01173478

[32] Held P, Owens GP, Anderson SE. The interrelationships among trauma-related guilt and shame, disengagement coping, and PTSD in a sample of treatment-seeking substance users. Traumatology. 2015;21(4):285–292. doi: 10.1037/trm0000050

[33] Iverson KM, Litwack SD, Pineles SL, Suvak MK, Vaughn RA, Resick PA. Predictors of intimate partner violence revictimization: The relative impact of distinct PTSD symptoms, dissociation, and coping strategies. Journal of Traumatic Stress. 2013;26(1):102–110. doi: 10.1002/jts.21781 23417878

[34] Faul F, Erdfelder E, Lang AG, Buchner A. G* Power 3: A flexible statistical power analysis program for the social, behavioral, and biomedical sciences. Behavior Research Methods. 2007;39(2):175–191. doi: 10.3758/bf03193146 17695343

[35] Tabachnick BG, Fidell LS, Ullman JB. Using multivariate statistics. Boston: Pearson; 2013. Vol. 6: pp. 497–516.

[36] Vrana S, Lauterbach D. Prevalence of traumatic events and post-traumatic psychological symptoms in a nonclinical sample of college students. Journal of Traumatic Stress. 1994;7: 289–302. doi: 10.1007/BF02102949 8012748

[37] Chen L, Jia S, Li P, Shi Z, Li Y. Experiences and coping strategies of somatic symptoms in patients with depressive disorder: A qualitative study. Archives of Psychiatric Nursing. 2022;38:6–13. doi: 10.1016/j.apnu.2022.01.004 35461645

[38] Selim A, Saad Salem S, Albasher N, Bakrmom G, Alanzi S, Jradi H. Irritable bowel syndrome and coping strategies: a cross-sectional study for identifying psychological alarms and factors related to coping in Riyadh, Saudi Arabia. Clinical Nursing Research. 2022;31(1):144–154. doi: 10.1177/10547738211020437 34056933

[39] Freyd JJ. A plea to university researchers. Journal of Trauma & Dissociation. 2012;13(5):497–508. doi: 10.1080/15299732.2012.708613 22989239

[40] Rocque R, Leanza Y. A systematic review of patients’ experiences in communicating with primary care physicians: Intercultural encounters and a balance between vulnerability and integrity. PloS ONE. 2015;10(10):e0139577. doi: 10.1371/journal.pone.0139577 26440647 PMC4594916

[41] Raja S, Rabinowitz EP, Gray MJ. Universal screening and trauma informed care: Current concerns and future directions. Families, Systems, & Health. 2021 Sep;39(3):526. doi: 10.1037/fsh0000585 33983761

[42] Lovell RC, Greenfield D, Johnson G, Eljiz K, Amanatidis S. Optimising outcomes for complex trauma survivors: assessing the motivators, barriers and enablers for implementing trauma informed practice within a multidisciplinary health setting. BMC Health Services Research. 2022 Apr 2;22(1):434. doi: 10.1186/s12913-022-07812-x 35366859 PMC8975732

